# Real‐world persistence of multiple sclerosis disease‐modifying therapies

**DOI:** 10.1111/ene.16289

**Published:** 2024-04-03

**Authors:** Emma C. Tallantyre, Ruth Dobson, Joseph L. J. Froud, Frederika A. St John, Valerie M. Anderson, Tarunya Arun, Lauren Buckley, Nikos Evangelou, Helen L. Ford, Ian Galea, Sumi George, Orla M. Gray, Aimee M. Hibbert, Mo Hu, Stella E. Hughes, Gillian Ingram, Seema Kalra, Chia‐Hui E. Lim, Joela T. M. Mathews, Gavin V. McDonnell, Naomi Mescall, Sam Norris, Stephen J. Ramsay, Claire M. Rice, Melanie J. Russell, Marianne J. Shawe‐Taylor, Thomas E. Williams, Katharine E. Harding, Neil P. Robertson

**Affiliations:** ^1^ Division of Psychological Medicine and Clinical Neurosciences Cardiff University Cardiff UK; ^2^ Department of Neurology University Hospital of Wales Cardiff UK; ^3^ Preventive Neurology Unit, Wolfson Institute of Population Health Queen Mary University London London UK; ^4^ Department of Neurology, Royal London Hospital Barts Health NHS Trust London UK; ^5^ Postgraduate Department St Thomas' Hospital London UK; ^6^ Department of Neuroscience University Hospitals Coventry and Warwickshire Coventry UK; ^7^ Department of Neurology, Southmead Hospital North Bristol NHS Trust Bristol UK; ^8^ Nottingham Centre for Multiple Sclerosis and Neuroinflammation, Queen's Medical Centre University Hospitals NHS Trust Nottingham UK; ^9^ University of Nottingham Nottingham UK; ^10^ Centre for Neurosciences, Leeds Teaching Hospitals NHS Trust Leeds General Infirmary Leeds UK; ^11^ Faculty of Medicine and Health University of Leeds Leeds UK; ^12^ Clinical Neurosciences, Clinical and Experimental Sciences, Faculty of Medicine University of Southampton Southampton UK; ^13^ Department of Neurology, Wessex Neurological Centre University Hospital Southampton NHS Foundation Trust Southampton UK; ^14^ Department of Neurology Ulster Hospital Dundonald UK; ^15^ Department of Neurology Swansea University Health Board Swansea UK; ^16^ MS Clinic Belfast City Hospital Belfast UK; ^17^ Neurology Department University Hospital North Midlands NHS Trust Stoke‐on‐Trent UK; ^18^ Barts Health NHS Trust London UK; ^19^ Queen Square Multiple Sclerosis Centre, Department of Neuroinflammation University College London London UK; ^20^ Aneurin Bevan University Health Board, Department of Neurology Royal Gwent Hospital Newport UK; ^21^ Transplantation Sciences, Bristol Medical School University of Bristol Bristol UK; ^22^ Faculty of Brain Sciences, Queen Square Institute of Neurology University College London London UK

**Keywords:** disease‐modifying therapy, multiple sclerosis, persistence, treatment

## Abstract

**Background and purpose:**

Treatment persistence is the continuation of therapy over time. It reflects a combination of treatment efficacy and tolerability. We aimed to describe real‐world rates of persistence on disease‐modifying therapies (DMTs) for people with multiple sclerosis (pwMS) and reasons for DMT discontinuation.

**Methods:**

Treatment data on 4366 consecutive people with relapse‐onset multiple sclerosis (MS) were pooled from 13 UK specialist centres during 2021. Inclusion criteria were exposure to at least one MS DMT and a complete history of DMT prescribing. PwMS in blinded clinical trials were excluded. Data collected included sex, age at MS onset, age at DMT initiation, DMT treatment dates, and reasons for stopping or switching DMT. For pwMS who had received immune reconstituting therapies (cladribine/alemtuzumab), discontinuation date was defined as starting an alternative DMT. Kaplan–Meier survival analyses were used to express DMT persistence.

**Results:**

In 6997 treatment events (1.6 per person with MS), median time spent on any single maintenance DMT was 4.3 years (95% confidence interval = 4.1–4.5 years). The commonest overall reasons for DMT discontinuation were adverse events (35.0%) and lack of efficacy (30.3%). After 10 years, 20% of people treated with alemtuzumab had received another subsequent DMT, compared to 82% of people treated with interferon or glatiramer acetate.

**Conclusions:**

Immune reconstituting DMTs may have the highest potential to offer a single treatment for relapsing MS. Comparative data on DMT persistence and reasons for discontinuation are valuable to inform treatment decisions and in personalizing treatment in MS.

## INTRODUCTION

**FIGURE 1:**
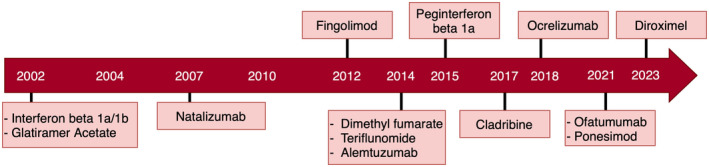
Schematic diagram illustrating the UK year of approval of each disease‐modifying therapy during the study period.

The past 20 years have seen the emergence of more than 15 disease‐modifying therapies (DMTs) for multiple sclerosis (MS; Figure 1). Phase 3 clinical trials provide evidence for DMT efficacy and tolerability in selected populations. However, real‐world effectiveness is highly dependent on treatment compliance, side effects, and patient/clinician concerns regarding longer term safety, all of which impact on overall treatment persistence. Persistence on treatment is the total time exposed to a treatment before either treatment escalation/de‐escalation, horizontal switching, or cessation. Clinical trials and their long‐term extension studies provide some information about persistence on individual DMTs, but do not fully reflect real‐world experience, because they are performed under controlled conditions in tightly defined populations with specific clinical characteristics [1].

A common question from people with MS (pwMS) when initiating DMT is “How long will I be taking this medication?”; there is currently no evidence‐based answer. Treatment interruption to switch from one DMT to another introduces a theoretical risk of MS reactivation. Cessation of certain DMTs can also prompt rebound disease activity [2, 3]. Furthermore, switching DMTs can be a negative emotional experience due to uncertainty around efficacy, change in administration routine, and risk profile [4]. The two most common reasons that prompt DMT switching at a population level appear to be lack of efficacy and side effects [5, 6, 7, 8, 9, 10, 11], although there are limited comparative data on the most common reasons for stopping individual DMTs.

Real‐world studies of persistence can be based on insurance claims data, registry data, or retrospective chart reviews. Claims‐based studies have mostly focussed on injectable (beta‐interferon [IFN] and glatiramer acetate [GA]) or oral DMTs, whereas registry studies tend to offer broader comparisons between DMTs [12]. Studies have reported a wide range of estimates for 12‐month persistence on any single DMT, ranging from 45% to 97%, and falling to 50%–60% by years 2–4 [5, 6, 13, 14, 15, 16, 17, 18, 19, 20, 21, 22, 23, 24, 25]. Very few data are available on longer term DMT persistence. The wide variance in estimates of persistence likely reflects differences in safety, tolerability, and efficacy profiles of individual DMTs, along with variations in practice between different centres and neurologists.

Higher persistence has been reported for oral than injectable (IFN and GA) DMTs [18, 19, 20, 22, 25], but few studies have compared the full range of currently available DMTs. Alemtuzumab and cladribine are induction (immune reconstitution) DMTs. Their mode of action and recommended treatment schedule require them to be considered differently from maintenance (continuously dosed) DMTs [26]. Studies have shown variable but often durable effects of these treatments, following initial and subsequent courses [27, 28]. The Big MS Data Network provided some comparative data on DMT usage in more than 110,000 pwMS treated up to 2016. Although persistence rates could not be reported, relatively stable rates of discontinuation over time were observed, with higher discontinuation rates seen for IFN and GA than natalizumab or fingolimod [7]. In addition, MS‐BASE data from 2023 demonstrated that cladribine use was associated with higher persistence than other oral DMTs [12].

A detailed contemporary understanding of real‐world DMT persistence in MS, as well as the reasons for stopping or switching treatments, has the potential to inform treatment algorithms and guide patient counselling. We aim to describe real‐world DMT persistence and reasons for stopping DMTs in a large multicentre cohort recruited from across the UK.

## METHODS

### Data collection

As part of the UK MS Trials and Registries consortium, regional MS centres were invited to provide data on pwMS exposed to DMT, irrespective of disease duration. Collation of data from numerous MS centres was used to mitigate potential biases arising due to variations in local prescribing habits. Three centres provided comprehensive data from local disease or DMT registries, which have been embedded in routine clinical practice for variable durations (Barts Health NHS Trust [London], since 2004; University Hospital of Wales [Cardiff] since 1999; Leeds Teaching Hospitals NHS Trust since 1995). Ten centres (Belfast City Hospital, Southmead Hospital [Bristol], University Hospitals of Coventry and Warwickshire, Royal Gwent Hospital [Newport], Nottingham University Hospitals NHS Trust, Southampton NHS Foundation Trust, Royal Stoke University Hospital, Swansea Bay University Health Board, Ulster Hospital [Belfast], and University College London NHS Foundation Trust) provided data from retrospective review of clinical records.

Inclusion criteria were (i) relapse‐onset MS, (ii) having received at least one DMT, and (iii) availability of complete records of DMT prescriptions for the entire duration of their MS. pwMS enrolled in blinded randomized treatment trials were excluded. At each centre, data were collected from local databases where available, supplemented by case note review to provide information on age at MS symptom onset, sex, sequential start and stop dates of all DMTs ever received, reasons for discontinuing any DMT, and last known follow‐up date. Centres were asked to contribute data on at least 100 consecutive patients seen from 1 January 2019. We aimed for at least 100 pwMS per DMT group, because that would provide 95% confidence of detecting a DMT discontinuation rate of 50%, with 10% margin of error. A dedicated data capture form was used by each centre to ensure data validity. Investigators were asked to allocate the main reasons for DMT discontinuation according to the following eight categories: (i) adverse event; (ii) high risk of adverse event (patient judged to be at unacceptably increased risk of adverse event e.g. high JCV index indicating higher risk of progressive multifocal leukoencephalopathy [PML]); (iii) disability progression; (iv) drug holiday (clinician/patient initiates a period off all treatment to determine whether to continue); (v) lack of efficacy (evidence of either relapse or subclinical magnetic resonance imaging activity); (vi) patient choice; (vii) family planning (including both patients who became pregnant on a DMT and those who elected to stop the DMT to try to conceive); and (viii) other, or reason was documented as unknown. Data were collated at each centre between August and November 2021, and anonymized data were transferred for analysis.

This study was approved by Health and Care Research Wales/the Health Research Authority (22/HCRW/0006) and was not reviewed by a research ethics committee because the research was limited to using previously collected, nonidentifiable information.

### Data analysis

We aimed to address two key aims. Our primary aim was to calculate individual DMT persistence. A secondary aim was to report the most common reasons for discontinuation of each DMT, along with patterns of clinical practice around DMT switching.

Descriptive statistics were used to explore patterns of DMT prescribing within our cohort. To address our first aim, DMT persistence was defined as the length of time a patient remained on a single DMT. To calculate median DMT persistence, we used Kaplan–Meier survival analysis according to DMT product. DMT data in this analysis were censored at time of DMT cessation or at date of last known follow‐up, depending on which came first. Where second or subsequent DMTs were started during follow up, these were included in survival analysis as a new DMT start. Interruptions in treatment for any reason that lasted <4 weeks and were followed by recommencement with the same product were considered uninterrupted treatment. Treatment interruptions of >4 weeks followed by recommencement of the same product were considered a cessation, and the recommencement a new DMT start. All IFN products were condensed into a single DMT category.

Alemtuzumab and cladribine administration is intermittent. For the purposes of addressing our primary question regarding persistence on a single DMT, in those who completed their first treatment course of alemtuzumab or cladribine (year 1 plus year 2), persistence was taken as the time to first DMT switch or time to last known follow‐up if no subsequent DMT had been prescribed. Those who failed to complete the first treatment course were marked as having stopped and reason for discontinuation was recorded.

To address our secondary aims, we reported reasons for DMT discontinuation according to the eight categories listed above. We calculated the mean interval between stopping one maintenance DMT and starting the next DMT.

## RESULTS

**FIGURE 2:**
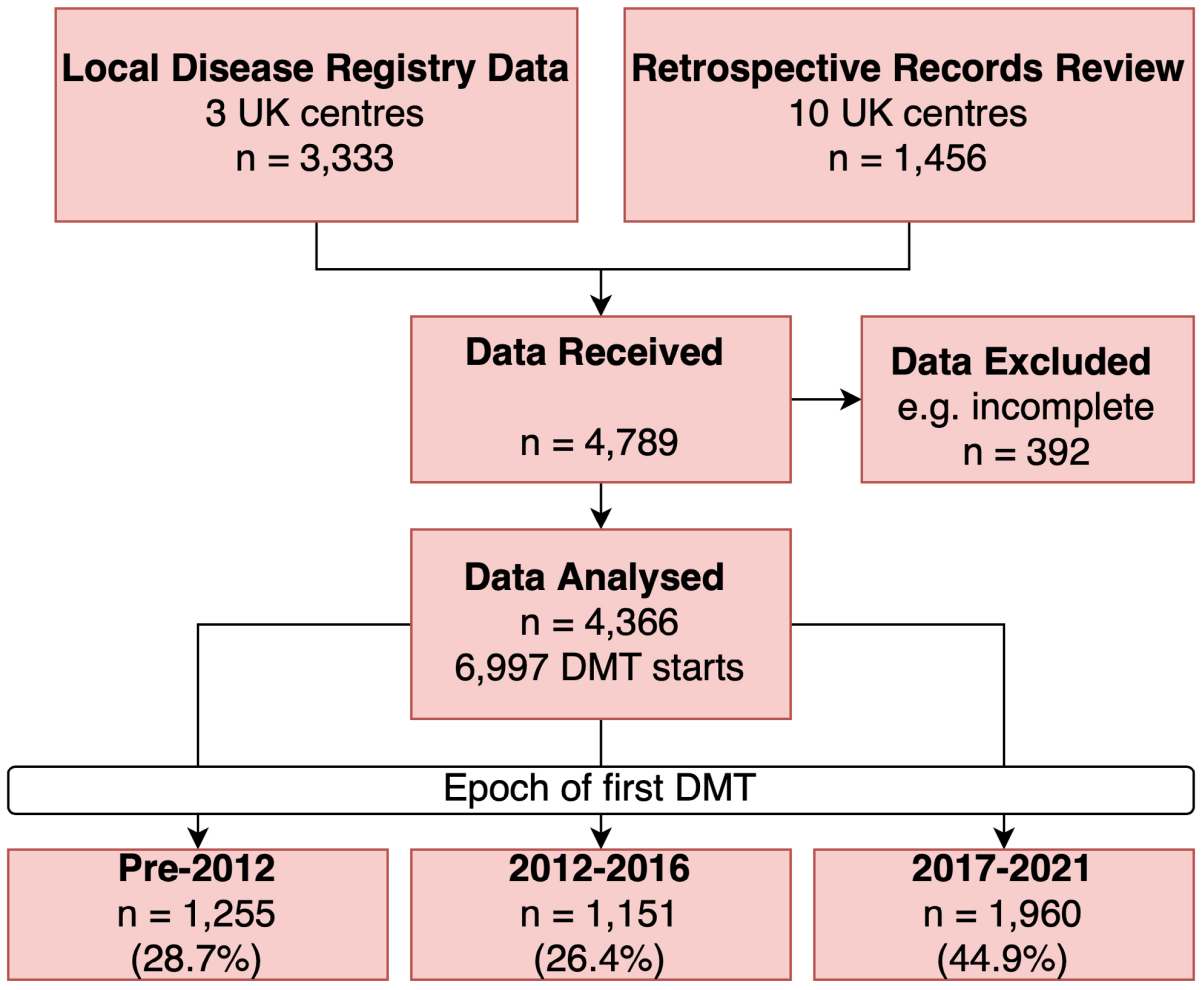
Study design. DMT, disease‐modifying therapy.

Data were available on 4789 pwMS with relapse‐onset MS who had received at least one DMT. A total of 423 (8.8%) case records were excluded because of missing data on DMT start/stop date(s) or commencement of DMT after date of last known follow‐up (Figure 2). The remaining 4366 pwMS were included in the analysis. Of these, 3152 (72%) were female; mean age at first DMT prescription was 37.6 years (range = 7–76). A total of 1255 (28.7%) received their first DMT pre‐2012, 1151 (26.4%) between 2012 and 2016, and 1960 (44.9%) during 2017–2021. Clinical–demographic characteristics of the cohort are shown in Table 1, and year of DMT approval in the UK is shown in Figure 1. As expected, the two centres with the longest running patient registries (Cardiff and Leeds) contributed most to the pre‐2012 dataset. Mean follow‐up time from first DMT was 3.8 years (SD = 3.8, median = 2.8 years), during which time pwMS received a mean of 1.6 (range = 1–5) DMTs. There were a total of 6997 DMT starts and 3324 DMT stops during the study period.

**TABLE 1:** Clinicodemographic characteristics of the cohort.

Characteristic	Total cohort, *N* = 4366
Participants per site, *n*	Barts 1016
	Belfast 196
	Bristol 99
	Cardiff 802
	Leeds 1169
	Newport 263
	Nottingham 105
	Southampton 100
	Stoke 94
	Swansea 230
	UCL Hospital 100
	University Hospital Coventry 94
	Ulster 98
Sex, female, *n* (%)	3152 (72%)
Age at first DMT, years, mean (SD)	37.6 (10.2)
Disease duration at first DMT, years, mean (SD)	6.3 (6.6)
Number of DMTs received at last follow‐up, mean, (SD)	1.6 (0.9)

Abbreviations: DMT, disease‐modifying therapy; UCL, University College London.

### DMT persistence

**FIGURE 3:**
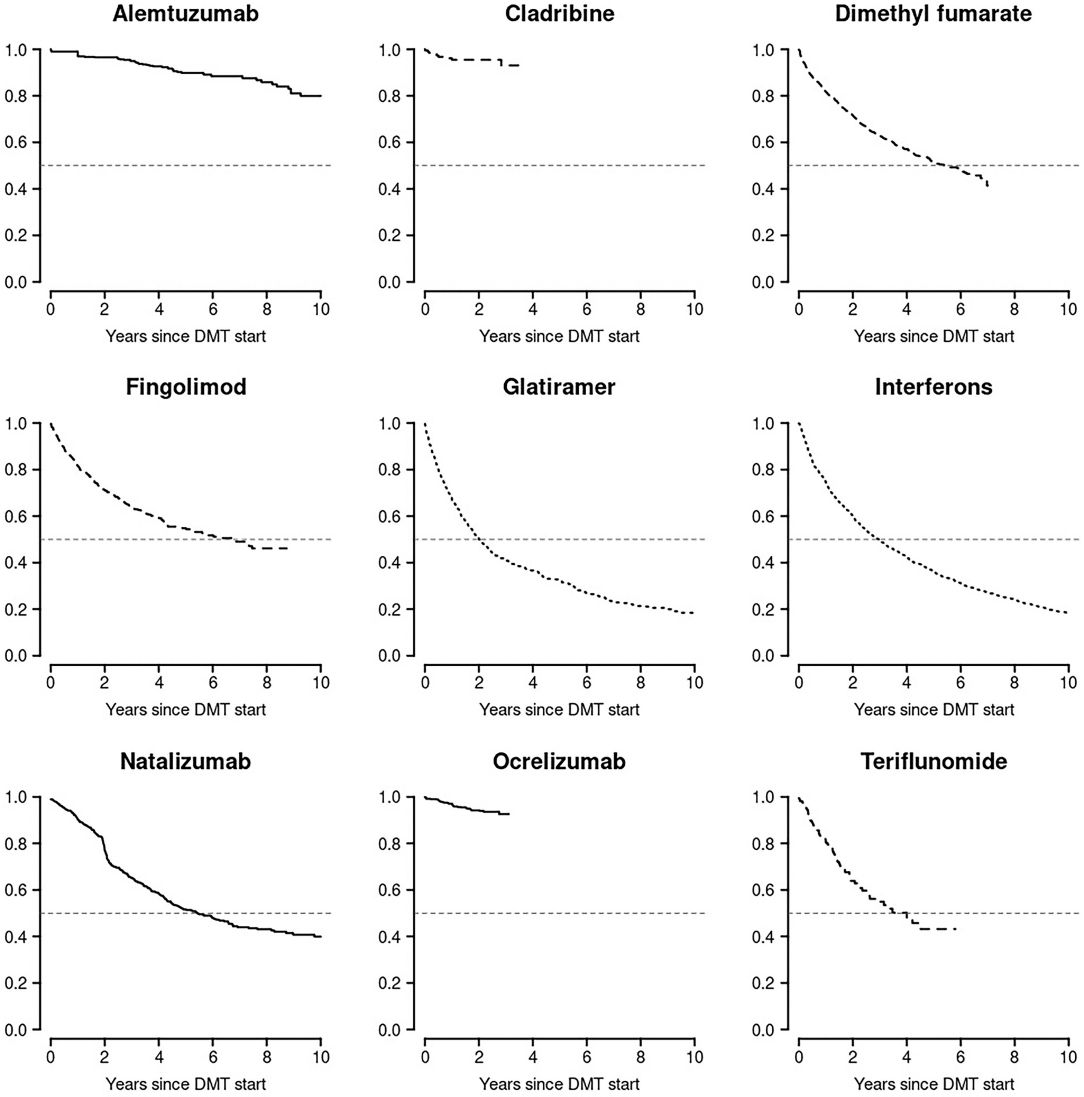
Kaplan–Meier survival curves illustrating cumulative persistence on disease‐modifying therapy (DMT) from time of commencement of treatment, by DMT.

The median time spent on any single maintenance DMT (i.e., excluding alemtuzumab and cladribine) was 4.3 years (95% confidence interval [CI] = 4.1–4.5 years). Treatment persistence per DMT is shown in Figure 3 and Table 2. Of the maintenance DMTs, ocrelizumab demonstrated the highest 2‐year persistence rate (94.2%). The 5‐year and 10‐year persistence of ocrelizumab could not be calculated due to its relative recency to market.

**TABLE 2:** Prescribing patterns and persistence of DMTs.

DMT	DMT naïve, *n* (%)	Overall DMT stops, *n* (%)	2‐year persistence (95% CI)	5‐year persistence (95% CI)	10‐year persistence (95% CI)
Alem, *n* = 504	362 (72%)	59 (12%)	96.5% (94.9–98.2) (*n* = 464)	89.8% (86.8–92.9) (*n* = 219)	80.0% (74.0–86.4) (*n* = 76)
Clad, *n* = 200	149 (75%)	9 (5%)	95.5% (92.4–98.6) (*n* = 142)	–	–
DMF, *n* = 1508	956 (63%)	608 (40%)	71.6% (69.2–74.0) (*n* = 863)	51.4% (48.4–54.6) (*n* = 262)	–
Fingo, *n* = 500	81 (16%)	213 (43%)	71.4% (67.5–75.5) (*n* = 332)	54.8% (50.2–59.7) (*n* = 149)	–
GA, *n* = 697	396 (57%)	512 (73%)	50.5% (46.8–54.4) (*n* = 313)	32.4% (28.8–36.4) (*n* = 149)	18.1% (14.9–22.0) (*n* = 55)
IFN, *n* = 1829	1420 (78%)	1500 (82%)	60.1% (57.9–62.4) (*n* = 1044)	36.1% (33.9–38.5) (*n* = 568)	18.1% (16.2–20.1) (*n* = 226)
Nat, *n* = 745	436 (59%)	314 (42%)	77.3% (74.1%–80.6%) (*n* = 457)	51.6% (47.5–56.1) (*n* = 206)	35.1% (29.3–42.1) (*n* = 48)
Ocr, *n* = 853	520 (61%)	43 (5%)	94.2% (92.4%–96.1%) (*n* = 434)	–	–
Ter, *n* = 161	46 (29%)	63 (39%)	63.9% (56.0%–72.8%) (*n* = 68)	43.2% (33.3–56.1) (*n* = 18)	–

*Note*: Numbers in the first column represent the number of DMT starts (*n* = 6997). One person with MS may have had several sequential treatments.

Abbreviations: Alem, alemtuzumab; CI, confidence interval; Clad, cladribine; DMF, dimethyl fumarate; DMT, disease‐modifying therapy; Fingo, fingolimod; GA, glatiramer acetate; IFN, beta‐interferon; Nat, natalizumab; Ocr, ocrelizumab; Ter, teriflunomide.

The immune reconstitution DMTs also demonstrated high rates of persistence. The 2‐year persistence on alemtuzumab and cladribine was 96.5% and 95.5%, respectively. The rates of persistence on alemtuzumab (completed the initial 2‐year course and did not receive any other subsequent DMT) were 89.8% at 5 years and 80.0% at 10 years. The 5‐year and 10‐year persistence of cladribine could not be calculated due to its relative recency to market. To further explore the apparent long‐term persistence of immune reconstituting DMTs, we also explored the rates of ever switching from an immune reconstituting DMT (alemtuzumab or cladribine) versus all other DMTs. Of 704 recorded first courses of alemtuzumab (*n* = 504) or cladribine (*n* = 200), only 46 (6.5%) pwMS ever received an alternative subsequent DMT (mean follow‐up duration = 4.3 years). Of 6293 recorded other DMT starts (excluding alemtuzumab and cladribine), 2554 (40.6%) ultimately switched to an alternative DMT (mean follow‐up duration = 3.2 years).

Natalizumab, fingolimod, and dimethyl fumarate all demonstrated moderate persistence at 2 years (77.3%, 71.4%, and 71.6%, respectively), which fell at 5 years to 51.6%, 54.8%, and 51.4%, respectively. There was a noticeable inflection point for reduction of persistence on natalizumab at 2 years (Figure 3). Teriflunomide had slightly lower persistence: 63.9% at 2 years and 43.2% at 5 years. IFN and GA demonstrated the lowest persistence. Rates of persistence on IFN were 60.1% at 2 years and 36.1% at 5 years. Rates of persistence on GA were 50.5% at 2 years and 32.4% at 5 years. Both injectable DMTs shared a persistence rate of only 18.1% at 10 years.

### Treatment line

Treatment persistence was 5.7 years for first‐line DMTs (95% CI = 5.3–6.0 years), 4.3 years for second‐line DMTs (95% CI = 4.0–4.9 years), and 3.9 years for third‐line DMTs (95% CI = 3.4–4.7 years), and the difference remained significant after adjustment for calendar year of DMT start (*p* < 0.0001). For the group of patients who had a moderate‐efficacy DMT as first‐line treatment and a high‐efficacy DMT as second‐line treatment (*n* = 312), persistence on the first‐line DMT was 1.52 years (95% CI = 1.30–1.8 years), compared to 7.3 years (lower 95% confidence limit = 5.93) on the second‐line DMT (*p* < 0.0001). Patient characteristics by first DMT are summarized in Table S1.

### Reasons for DMT discontinuation

**FIGURE 4:**
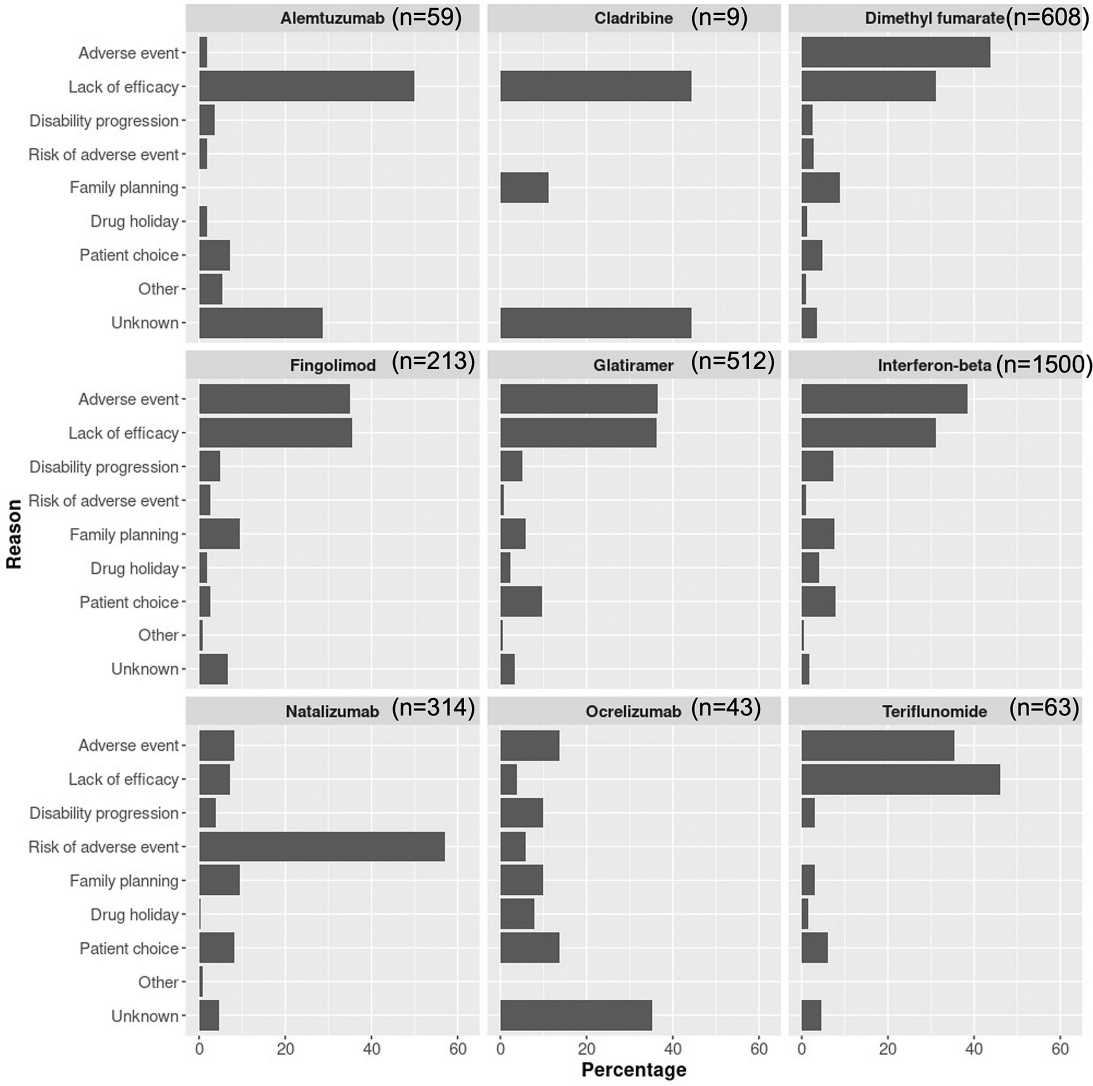
Bar chart showing the percentage of reasons for discontinuation of disease‐modifying therapy (DMT), by DMT.

The commonest reasons for DMT discontinuation overall were adverse events (1170 of 3324 DMT stops; 35.2%) and lack of efficacy (1012/3324; 30.4%), which remained stable over treatment epochs (Table 3). Reasons for stopping individual DMTs varied considerably (Figure 4). In the low number of cases (*n* = 46) where alemtuzumab or cladribine were followed by another DMT, the most common reason was lack of efficacy (48%). The most common reasons for discontinuation of ocrelizumab (*n* = 43) were adverse events (15%), patient choice (15%), disability progression (10%), and family planning (10%). Lack of efficacy and adverse events were the major reasons underlying discontinuation of dimethyl fumarate, fingolimod, teriflunomide, IFN, and GA. However, the most common reason for stopping natalizumab (58%) was increased risk of an adverse event (PML).

**TABLE 3:** Reasons for stopping DMT according to epoch of commencement.

Reason for stopping, *n* = 3362 DMT stops	Date of DMT commencement		
	Pre‐2012	2012–2016	2017–2021
Adverse events	444 (33%)	439 (35%)	287 (37%)
Disease progression	133 (10%)	49 (4%)	14 (2%)
Drug holiday	60 (5%)	18 (1%)	14 (2%)
Increased risk of adverse event	66 (5%)	102 (8%)	60 (8%)
Lack of efficacy	382 (29%)	410 (32%)	220 (29%)
Other	9 (1%)	7 (1%)	9 (1%)
Patient choice	94 (7%)	100 (8%)	49 (6%)
Pregnancy planning	105 (8%)	97 (8%)	56 (7%)
Unknown	36 (3%)	44 (3%)	58 (8%)

Abbreviation: DMT, disease‐modifying therapy.

The median off‐treatment interval in those pwMS who stopped a maintenance DMT and subsequently started another DMT was 68 (interquartile range = 15–161) days (excluding ocrelizumab due to its infrequent dosing schedule). There was variability in off‐treatment interval according to DMT (Table 4). Overall, the DMTs that were most often used following discontinuation of natalizumab due to increased risk of adverse events (*n* = 181) were fingolimod (45%) and ocrelizumab (34%).

**TABLE 4:** Time from stop of DMT to commencement of next DMT according to drug.

DMT	Number of DMT stops	Median (IQR) interval to next DMT after stopping, days
Dimethyl fumarate	503	68 (23–175)
Fingolimod	182	84 (37–183)
Glatiramer	405	37 (5–158)
Interferon	1112	72 (24–294)
Natalizumab	273	80 (42–165)
Teriflunomide	54	58 (10–139)

Abbreviations: DMT, disease‐modifying therapy; IQR, interquartile range.

## DISCUSSION

Understanding real‐world DMT persistence is helpful in informing clinical decisions and counselling of pwMS. Persisting on a maintenance DMT contributes to overall treatment effectiveness and avoids complications associated with DMT cessation such as rebound of MS activity and the risk of breakthrough disease activity while there is interruption between sequential DMTs. For some pwMS, receiving a single DMT for MS reduces anxiety associated with switching DMT products [4]. For the first time, in this large multicentre study of real‐world data, we provide comparative persistence rates for MS DMTs spanning all current mechanisms of action and provide detailed data on reasons for discontinuation.

A novel finding from this study was that the pwMS with the highest chance of a single DMT intervention for MS were those who received immune reconstituting therapy (alemtuzumab or cladribine). The results are mainly driven by alemtuzumab, because cladribine has been more recent to the market. The 2‐year, 5‐year, and 10‐year persistence on alemtuzumab was consistently ≥80%, in >500 pwMS who were mostly DMT‐naïve. It is important to note that persistence on immune reconstituting therapies had to be defined slightly differently in this study (time to next DMT or most recent follow‐up), on account of differences in their schedule of administration. It follows that treatment‐associated adverse events following immune reconstituting DMTs such as secondary autoimmunity emerging months or years after alemtuzumab administration [29, 30] would not have been captured in our analysis. This is important to note, because during postmarketing use of alemtuzumab, some rare, serious, sometimes fatal adverse events have been reported [31]. Likewise, emergence of new disease activity following immune reconstituting therapies may have resulted in retreatment with the same product (which has been shown to occur in 35%–45% recipients of alemtuzumab at 6 years) [29, 30], but this would still fulfil a definition of persistence in our study. With these caveats in mind, pwMS may still benefit from knowing that induction therapies appear to be associated with a low chance of needing to subsequently switch DMTs in real‐world practice.

To our knowledge, this is the first time that long‐term persistence of immune reconstituting therapies has been explored and compared with maintenance therapies. Data from CARE‐MS I/II extension studies also showed >90% retention rates on alemtuzumab at 5 years [32, 33], but caveats apply regarding extrapolation of this to the real world. Similarly, a comparison study of several DMTs by MS‐Base suggested high persistence on alemtuzumab, although follow‐up duration was short; cladribine and ocrelizumab were not studied [7]. Another MS‐Base study comparing oral DMTs showed 95% persistence on cladribine after a mean of 1.14 years of follow‐up [12]. A real‐world study of 124 pwMS who had at least 24 months of follow‐up after first cladribine dose found that <5% started an alternative DMT [34].

Ocrelizumab was also shown to have high rates of persistence. Although its relatively recent licensing meant that 5‐year persistence could not be calculated, our results suggest that ocrelizumab is well tolerated in years 1–3. This is in keeping with long‐term extension data for anti‐CD20 DMTs, suggesting >85% persistence at 4–5 years [35, 36]. Family planning was among the most common reasons cited in the small number of pwMS who stopped ocrelizumab. However, growing safety data may support off‐license use of ocrelizumab as part of pregnancy planning, with the potential to shorten the recommended 6‐month washout prior to trying to conceive [37]. The growing recognition that treatment‐related complications such as hypogammaglobulinaemia and infection may be cumulative during anti‐CD20 treatment also suggests that data on longer term persistence on anti‐CD20 DMTs (including the newer agents ofatumumab and ublituximab) will be valuable and informative [38]. In contrast, data suggesting that anti‐CD20s may have some immune reconstituting effect may mean that future trials are needed to explore the utility of these DMTs in an immune reconstituting schedule, more closely resembling that of alemtuzumab and cladribine [26].

Natalizumab demonstrated moderately high 2‐year persistence but showed lower persistence at 5 years, which was largely accounted for by expected discontinuations due to increased risk of adverse events. The risk mitigation programme for natalizumab requires pwMS and clinicians to calculate PML risk for each year of treatment, informing the risk–benefit profile of continued treatment. The increase in risk beyond the second year of treatment seemed evident in this cohort, because the inflection point of persistence on natalizumab was apparent at year 2. The most common DMTs to be used after natalizumab (when discontinued due to increased risk of adverse events) were fingolimod and ocrelizumab, in keeping with other reports [39]. We did not have sufficient power to explore patterns of switching over epochs of time. Family planning was relatively rarely cited as a reason to discontinue natalizumab, in line with its position as a high‐efficacy DMT that has a relatively favourable safety profile in pregnancy [37].

Persistence on fingolimod and dimethyl fumarate were similar in our cohort. The 2‐year persistence rates of approximately 70% for both are broadly in line with other estimates from the literature [12, 20, 21, 22, 40, 41]. Slightly more than one half of people commenced on these two DMTs persisted on them at 5 years. Adverse events appeared to account for a slightly greater proportion of discontinuations of dimethyl fumarate versus fingolimod. Slightly lower persistence rates were seen for teriflunomide, with <50% pwMS remaining on this DMT by 5 years. Lack of efficacy was cited as the most common reason for discontinuing teriflunomide, which appears to be in line with other work [42]. The persistence rates for injectable DMTs (IFN and GA) were lower than oral DMTs, in line with data from other studies that have compared these two approaches [18, 19, 20, 21, 22, 40, 41, 43]. Lack of efficacy and adverse events were the major contributors to discontinuation.

This work is subject to some further limitations. Our study combined data from local registries and retrospective chart review and therefore is more prone to bias, including recall bias, compared to a common registry platform with prospective data entry. We were unable to adjust for baseline patient characteristics given the limitations of available data, range of data sources used, and risk of inadvertently introducing bias through nonrandom missingness. Ofatumumab, ponesimod, and ublituximab were not widely available in the UK during this study period and were therefore not captured in this dataset. Siponimod is only approved for use in secondary progressive MS in the UK, and ocrelizumab is the only approved drug for people with primary progressive MS, so neither of these prescribing patterns were included in this analysis. The long‐term persistence of recently approved DMTs is difficult to assess. Likewise, the evolving DMT landscape, according to drug licensing and local approvals over time, inevitably influences prescribing practice and possibly also persistence. For maintenance DMTs, we did not attempt to measure compliance (taking the medication as prescribed), which was presumed.

## CONCLUSIONS

Treatment persistence is highly relevant in MS. For many pwMS, receiving a single DMT product minimizes anxiety associated with switching DMT products [4]. For maintenance DMTs, persistence on one therapy is likely to contribute to real‐world effectiveness and reduce the risk of disease reactivation on discontinuing or switching. Our large comparative, real‐world study on DMT persistence provides novel data to inform counselling of pwMS including the common question, “How long will I stay on this therapy?”. Immune reconstituting DMTs appear to demonstrate high potential for offering a single, durable treatment for relapsing multiple sclerosis.

## AUTHOR CONTRIBUTIONS

**Emma C. Tallantyre:** Conceptualization; investigation; methodology; writing – original draft. **Ruth Dobson:** Methodology; writing – review and editing; writing – original draft; investigation. **Joseph L. J. Froud:** Investigation; writing – review and editing; data curation. **Frederika A. St John:** Data curation; investigation; writing – review and editing. **Valerie M. Anderson:** Investigation; methodology; data curation; project administration; writing – review and editing. **Tarunya Arun:** Investigation; writing – review and editing. **Lauren Buckley:** Investigation; writing – review and editing. **Nikos Evangelou:** Investigation; writing – review and editing. **Helen L. Ford:** Investigation; writing – review and editing. **Ian Galea:** Investigation; writing – review and editing. **Sumi George:** Investigation; writing – review and editing. **Orla M. Gray:** Investigation; writing – review and editing. **Aimee M. Hibbert:** Investigation; writing – review and editing. **Mo Hu:** Investigation; writing – review and editing. **Stella E. Hughes:** Investigation; writing – review and editing. **Gillian Ingram:** Investigation; writing – review and editing. **Seema Kalra:** Investigation; writing – review and editing. **Chia‐Hui E. Lim:** Investigation; writing – review and editing. **Joela T. M. Mathews:** Investigation; writing – review and editing. **Gavin V. McDonnell:** Investigation; writing – review and editing. **Naomi Mescall:** Investigation; writing – review and editing. **Sam Norris:** Investigation. **Stephen J. Ramsay:** Investigation; writing – review and editing. **Claire M. Rice:** Investigation; writing – review and editing. **Melanie J. Russell:** Investigation; writing – review and editing. **Marianne J. Shawe‐Taylor:** Investigation; writing – review and editing. **Thomas E. Williams:** Investigation; writing – review and editing. **Katharine E. Harding:** Investigation; writing – review and editing; formal analysis; data curation; methodology. **Neil P. Robertson:** Investigation; conceptualization; methodology; validation; writing – original draft; writing – review and editing; supervision.

## CONFLICT OF INTEREST STATEMENT

C.M.R. has received research funding from Sanofi. E.C.T. has received honoraria for consulting work, or speaker fees, from Biogen, Janssen, Merck, Novartis, and Roche, and has received travel grants to attend or speak at educational meetings from Biogen, Merck, Roche, and Novartis. G.I. has received honoraria for consulting work, or speaker fees, from Biogen, Novartis, and Roche, and has received travel grants to attend or speak at educational meetings from Biogen, Merck, Roche, and Novartis. H.L.F. has received honoraria for consulting work and/or educational activities from Biogen, Merck, Novartis, Roche, Sanofi Genzyme, and Teva. I.G. has received a research grant from Merck‐Serono. J.T.M.M. has received honoraria for consulting work, or speaker fees, from Biogen, Celegene BMS, Janssen, Merck, Novartis, Roche, Sandoz, and Sanofi. She has received travel grants to attend or speak at educational meetings from Biogen, Janssen, Merck, Novartis, Roche, Sandoz, and Sanofi. K.E.H. has received honoraria for consulting work, or speaker fees, from Biogen, Merck, and Roche, and has received travel grants to attend or speak at educational meetings from Biogen, Merck, Roche, and Novartis. M.J.R. has received speaker fees from Biogen. O.M.G. has received honoraria as a consultant on scientific advisory boards for Genzyme, Biogen, Merck, Roche, and Novartis; has received travel grants from Biogen, Merck, Roche, Sanofi, and Novartis; has participated in clinical trials by Biogen; and has received research funding from Biogen. R.D. has received educational/personal honoraria (to her organization) from Roche, Novartis, Sandoz, Biogen, and Merck, and research funding from Biogen and Merck. S.E.H. has received honoraria for consulting work, or speaker fees, from Biogen, Merck, Novartis, and Roche. She has received travel grants to attend conferences from Biogen, Merck, Roche, and Novartis. S.K. has received honoraria for consulting work from Biogen and Novartis. She has received travel grants to attend or speak at educational meetings from Biogen and Novartis. T.A. has received honoraria for advisory work, speaker fees, or research grants from Biogen, Merck, Novartis, Roche, and Janssen. T.E.W. has received honoraria for educational talks from Novartis and Merck. None of the other authors has any conflict of interest to disclose.

## Supporting information


TABLE S1.

## Data Availability

The data that support the findings of this study are available on request from the corresponding author. The data are not publicly available due to privacy or ethical restrictions.
