# Supplementary material for: Real‐world persistence of multiple sclerosis disease‐modifying therapies
Source: Eur J Neurol. 2024 Apr 3;31(7):e16289. doi: 10.1111/ene.16289 (PMC11235620; doi:10.1111/ene.16289)
Supplement: Supplementary file 1 — TABLE S1. [file ENE-31-e16289-s001.docx]

**Supplementary Table 1 Demographic characteristics of people with MS at the time of commencing first DMT (n=4366)**

| **First DMT** | **Total number** | **Female** | **Mean age at DMT start (standard deviation)** | **Mean disease duration at DMT start (standard deviation)** |
| --- | --- | --- | --- | --- |
| Alemtuzumab | 362 | 257 (71%) | 34.4 years (8.6) | 3.5 years (3.6) |
| Cladribine | 149 | 108 (72%) | 39.6 years (12.6) | 5.3 years (6.2) |
| Dimethyl fumarate | 956 | 694 (73%) | 39.2 years (10.4) | 6.9 years (7.3) |
| Fingolimod | 81 | 55 (68%) | 40.0 years (10.9) | 9.9 years (10.9) |
| Glatiramer acetate | 396 | 303 (77%) | 37.1 years (9.2) | 6.2 years (6.1) |
| Interferon-β | 1420 | 1045 (74%) | 36.9 years (9.5) | 6.8 years (6.7) |
| Natalizumab | 436 | 305 (70%) | 35.2 years (10.4) | 5.6 years (5.8) |
| Ocrelizumab | 520 | 351 (68%) | 40.1 years (11.0) | 6.0 years (6.5) |
| Teriflunomide | 46 | 34 (74%) | 44.3 years (11.3) | 8.4 years (6.6) |
